# Low expression levels of ATM may substitute for *CHEK2 */*TP53 *mutations predicting resistance towards anthracycline and mitomycin chemotherapy in breast cancer

**DOI:** 10.1186/bcr3147

**Published:** 2012-03-15

**Authors:** Stian Knappskog, Ranjan Chrisanthar, Erik Løkkevik, Gun Anker, Bjørn Østenstad, Steinar Lundgren, Terje Risberg, Ingvil Mjaaland, Beryl Leirvaag, Hrvoje Miletic, Per E Lønning

**Affiliations:** 1 Section of Oncology, Institute of Medicine, University of Bergen, Jonas Lies vei 65, Bergen, 5020, Norway; 2 Department of Oncology, Haukeland University Hospital, Jonas Lies vei 65, Bergen, 5021, Norway; 3 Division of Surgery and Cancer Medicine, Department of Oncology, Oslo University Hospital, Ullernchausséen 70, Oslo, 0310, Norway; 4 Department of Oncology, Ullevaal University Hospital, Kirkeveien 166, Oslo, 0450, Norway; 5 Department of Oncology, St. Olav University Hospital, Olav Kyrres gate 17, Trondheim, 7006, Norway; 6 Department of Cancer Research and Molecular Medicine, Norwegian University of Science and Technology, Hogskoleringen 1, Trondheim, 7491, Norway; 7 Department of Oncology, University Hospital of Northern Norway and Institute of Clinical Medicine, University of Tromsø, Sykehusvegen 38, Tromsø, 9037, Norway; 8 Division of Hematology and Oncology, Stavanger University Hospital, Armauer Hansens vei 20, Stavanger, 4011, Norway; 9 Department of Pathology, Haukeland University Hospital, Jonas Lies vei 65, Bergen, 5021, Norway; 10 Department of Biomedicine, University of Bergen, Jonas Lies vei 91, Bergen, 5020, Norway; 11 Current address: Department of Immunology, Institute for Cancer Research, Oslo University Hospital Radiumhospitalet, Ullernchausséen 70, 0310 Oslo, Norway

## Abstract

**Introduction:**

Mutations affecting p53 or its upstream activator Chk2 are associated with resistance to DNA-damaging chemotherapy in breast cancer. ATM (Ataxia Telangiectasia Mutated protein) is the key activator of p53 and Chk2 in response to genotoxic stress. Here, we sought to evaluate ATM's potential role in resistance to chemotherapy.

**Methods:**

We sequenced *ATM *and assessed gene expression levels in pre-treatment biopsies from 71 locally advanced breast cancers treated in the neoadjuvant setting with doxorubicin monotherapy or mitomycin combined with 5-fluorouracil. Findings were confirmed in a separate patient cohort treated with epirubicin monotherapy. Each tumor was previously analyzed for *CHEK2 *and *TP53 *mutation status.

**Results:**

While *ATM *mutations were not associated with chemo-resistance, low ATM expression levels predicted chemo-resistance among patients with tumors wild-type for *TP53 *and *CHEK2 *(*P *= 0.028). Analyzing the ATM-chk2-p53 cascade, low ATM levels (defined as the lower 5 to 50% percentiles) or mutations inactivating *TP53 *or *CHEK2 *robustly predicted anthracycline resistance (*P*-values varying between 0.001 and 0.027 depending on the percentile used to define "low" ATM levels). These results were confirmed in an independent cohort of 109 patients treated with epirubicin monotherapy. In contrast, ATM-levels were not suppressed in resistant tumors harboring *TP53 *or *CHEK2 *mutations (*P *> 0.5).

**Conclusions:**

Our data indicate loss of function of the ATM-Chk2-p53 cascade to be strongly associated with resistance to anthracycline/mitomycin-containing chemotherapy in breast cancer.

## Introduction

Despite significant improvements in cancer therapy over the last decades, resistance towards chemotherapy remains the main obstacle to cure among patients suffering from solid tumors [1].

The molecular mechanisms causing chemo-resistance in breast cancer, as for most other cancer forms, are poorly understood. While Topoisomerase-II amplified tumors on average reveal enhanced anthracycline sensitivity compared to non-amplified tumors [2-5], lack of Topoisomerase-II expression may not explain anthracycline resistance.

p53, the tumor suppressor protein encoded by the *TP53 *gene, plays a key role with respect to apoptosis but also senescence, growth arrest and DNA repair [6,7]. Our group has previously linked mutations in *TP53*, (in particular those affecting the L2/L3 DNA binding domains), to resistance to anthracyclines and the related cytotoxic compound, mitomycin, in primary breast cancers [8-10]. However, the observation that some tumors harboring wild-type *TP53 *revealed resistance towards anthracycline therapy made us hypothesize this could be due to inactivation of other genes acting up- or downstream in the p53 functional pathway [11,12]. Excluding potential correlations between genetic and epigenetic alterations affecting cyclin-inhibitors and therapy resistance [8,13,14], subsequently, we detected non-sense mutations in the *CHEK2 *gene (coding for the Chk2 protein) in three patients with primary breast cancers revealing anthracycline resistance [8,15]. Chk2 phosphorylates p53 at Ser 20, inhibiting MDM2-p53 protein binding [16] but also at several sites located in the C-terminal domain of the p53 protein [17]. While Chk2 activates multiple downstream targets in addition to p53, and the p53 protein may be activated through multiple post-transcriptional events [17], the finding that *CHEK2 *mutations may substitute for *TP53 *mutations as a cause of chemo-resistance indicates Chk2 phosphorylation of the p53 protein to play a pivotal role executing cell death in response to anthracycline therapy in breast cancer.

Chk2 activation, in response to chemotherapy-induced double strand breaks, is mediated through the Ataxia Telangiectasia Mutated (ATM) protein which phosphorylates Chk2 at Thr 68 in response to DNA damage caused by cytotoxic compounds or ionizing radiation [18,19]. Further, ATM directly phosphorylates p53 at Ser 15, providing additional activation of p53 besides the Ser 20 phosphorylation [20,21].

Based on the evidence above, we hypothesized that loss of ATM function could be a cause of anthracycline resistance in breast cancers harboring wild-type *TP53 *and *CHEK2*. While low expression of ATM has been found associated with a poor prognosis among breast cancer patients harboring wild-type *TP53 *tumors treated with DNA-damaging chemotherapy [22], notably the direct effect of ATM status on response to anthracycline therapy (predictive value) has not been addressed previously.

In this study, we performed complete *ATM *gene sequencing and determined ATM mRNA levels in breast tumor samples selected based on *TP53 *and *CHEK2 *mutation status and clinical outcome. All samples were from primary breast cancers treated with pre-surgical ("neoadjuvant") therapy in controlled studies from which *TP53 *and *CHEK2 *gene mutation status had been previously characterized and the direct response to chemotherapy (doxorubicin, 5FU/mitomycin or epirubicin) as well as long-term outcome for each individual patient determined. We found low levels of tumor ATM expression to predict chemo-resistance in tumors wild-type for *TP53 *and *CHEK2 *but not in tumors harboring *TP53 *or *CHEK2 *mutations. Importantly, these findings were corroborated by the findings that low ATM expression signaled a poor prognosis among patients harboring *TP53 *and *CHK2 *wild-type tumors, contrasting an improved prognosis in tumors harboring *TP53*/*CHEK2 *mutations. These findings indicate the ATM-chk2-p53 cascade to be an important pathway executing drug-induced cell death in breast cancers *in vivo*.

## Materials and methods

### Patients

For this study we analyzed samples selected from three prospective studies [8-10]. The selection of tumors for this study was based on the patients' response to therapy and tumor *TP53 *and *CHEK2 *status, previously determined [8,10]. The rationale was to analyze an optimal number of "poor" and "good" responders and tumors mutated versus wild-type for *TP53*/*CHEK2*. Since the majority of patients displayed partial response upon treatment and wild-type status for *TP53 *and *CHEK2*, only a subgroup of these were included for ATM analyses and statistical comparisons. In contrast, we included all tumors harboring *TP53 */*CHEK2 *mutations and all tumors resistant to therapy.

The numbers of patients included in the different ATM analyses are listed in Table 1 with further details listed in Additional file 1 Table S1. Cohort 1 (*n *= 71) included a selection of 36 out of 91 patients enrolled in a prospective study exploring the mechanisms of resistance to treatment with doxorubicin in locally advanced breast cancer [10] as well as all 35 patients from a similar prospective study evaluating the mechanisms of resistance to 5-fluorouracil and mitomycin (FUMI) [9]. In this cohort of 71 tumors, material for DNA sequencing, mRNA expression analysis and MLPA copy number analysis was available from *n *= 70, *n *= 69 and *n *= 66 tumors, respectively. For the first part of the study, these patients were statistically evaluated as an exploratory data set (Cohort 1).

**Table 1:** Patients included in ATM analyses

Cohort		ATM coding region			ATM promoter		
**No**.	**Therapy**	**Mutations**	**mRNA levels**	**Copy no**.	**Mutations**	**Methylation**	**Survival**
		***n***	***n***	***n***	***n***	***n***	***n***

1	Doxorubicin	70	69	69	70	-	69
	FUMI						
							
2	Epirubicin	41	109^1^	-	41	109^1^	109^1^
							
3	Paclitaxel	38	114^2^	-	38	-	114^2^

^1 ^Among the 109 patients, two were omitted from statistical analyses as protocol violators.

^2 ^Among the 114 patients, 8 had non-evaluable response to therapy.

For validation purposes and inclusion of a group of patients treated with a non-anthracycline/mitomycin regimen, we analyzed samples from a study in which patients with primary breast cancers were randomized to pre-surgical treatment with epirubicin (Cohort 2; *n *= 109; validation cohort) versus paclitaxel (Cohort 3; *n *= 114; patients treated with a non-anthracycline-containing regimen) monotherapy [8,23]. Out of the 109 epirubicin treated patients, 2 were omitted from statistical analyses as protocol violators (one sarcomatoid and one stage II tumor) and from the 114 paclitaxel treated patients, 8 were omitted from statistical analyses due to non-evaluable response to therapy, in most cases due to early termination of treatment because of paclitaxel toxicity (for details, see [8]). Here, ATM expression was determined in the whole cohort, while ATM gene sequencing was performed in subgroups of 41 (Cohort 2) and 38 (Cohort 3) individuals.

Before commencing chemotherapy, each patient participating in one of these trials had an incisional tumor biopsy as described previously [9]. All tissue samples were snap-frozen immediately on removal in the surgical theater.

Each of the studies was approved by the local Ethical Committee, and all patients gave written informed consent.

### Nucleic acid isolation and cDNA synthesis

Genomic DNA was isolated from tumor biopsies using QIAamp DNA Mini kit (Qiagen, Chatsworth, CA, USA) according to the manufacturer's protocol. Total RNA was purified by Trizol (Life Technologies, Inc., Grand Island, NY, USA) extraction from snap-frozen tissue samples according to manufacturer's instructions. After extraction, the RNA was dissolved in DEPC treated ddH2O. First-strand cDNA synthesis was carried out using oligo-dT and Random Hexamer primers with the Transcriptor Reverse Transcriptase system (Roche, Basel, Switzerland) in accordance with the manufacturer's instructions.

### Mutation screening

Screening for mutation and small insertions/deletions was performed by PCR-amplification and subsequent sequencing of all the exons of the coding region of *ATM *[GenBank: NT 033899] as well as the previously described bidirectional promoter area [17]. All amplifications were performed using the Kod XL DNA polymerase system (Novagen, Madison, WI, USA) according to the manufacturer's instructions. Primers and thermal conditions for the primer annealing step are listed in Additional file 1 Table S2. Prior to sequencing, PCR products were purified using the ExoSAP-IT kit (GE Healthcare, Little Chalfont, UK) according to the manufacturer's instructions. Sequencing was done using BigDye version 1.1 cycle sequencing kit (ABI, Foster City, CA, USA) with specific forward or reverse sequencing primers, according to the manufacturer's instructions. Thermal conditions were 30 cycles of denaturation at 94°C for 10 seconds, annealing at 50°C for 5 seconds and elongation at 60°C for 4 minutes. Capillary electrophoresis, data collection and sequence analysis were performed on an automated DNA sequencer (ABI 3700). Resulting sequences from patient samples were analysed using Genebank accession u33841 as reference.

### Quantitative PCR

Quantitative PCRs were performed using specific Hydrolysis Probes targeting *ATM *on a Light Cycler 480 instrument (Roche). Reaction mixes were made according to the instructions from the manufacturer of the kit Lightcycler 480 ProbesMaster (Roche). Relative mRNA expressions were normalized to rpP2 gene expression in a two-colour duplex reaction. Primers/probes for detection of *ATM *(5'-*GCAGATGACCAAGAATGCAA*-3', 5'- *GGCCTGCTGTATGAGCAAAT*-3' and 6FAM-*TGGAAGAAGGCACTGTGCTCA*-BBQ) and *rpP2 *(5'-*gaccggctcaacaaggttat*-3', 5'-*ccccaccagcaggtacac*-3' and Cy5-*agctgaatggaaaaaacattgaagacgtc*-BBQ), were designed to be used in the same conditions of real-time PCR amplification. After initial denaturation at 95°C for five minutes, samples were run through 50 cycles of the following conditions: Denaturation for 10 seconds at 95°C and elongation at 55°C for 25 seconds. All reaction data were converted into relative concentrations through the use of an internal standard curve in each run. Each analysis was performed in triplicate.

### Copy number analyses

*ATM *gene copy numbers were determined by MLPA analysis using the SALSA MLPA P190 probemix (MRC-Holland, Amsterdam, The Netherlands) according to the manufacturer's instructions. Peak areas of all MLPA products resulting from *ATM *specific probes were normalized and compared to references as previously described [24].

### Promoter methylation analyses

Genomic DNA from patients was modified by bisulfite conversion, using the EZ DNA Methylation Gold Kit (Zymo Research, Irvine, CA, USA). Primers were used to specifically amplify methylated and unmethylated DNA immediately upstream of the ATM transcription start site (for primer sequences, see [25]). Methylation- and non-methylation-specific PCRs (MSP and USP) were performed using the AmpliTaq Gold DNA Polymerase system (Applied Biosystems, Foster City, CA, USA) in a 50 μl solution containing 1X PCR buffer, 1.5 mM MgCl_2_, 0.5 mM of each deoxynucleotide triphosphate, 0.2 μM of each primer and approximately 50 ng of modified genomic DNA. The thermocycling conditions for both the MSP and the USP were an initial 5 minutes of denaturation at 94°C followed by 35 cycles of 30 sec at 94°C, 30 sec at 57.6°C, and 30 sec at 72°C. Included in each run were a methylated control (CpGenome Universal Methylated DNA, Millipore, Billerica, MA, USA), an unmethylated control (modified DNA from healthy donors) and a negative control (water). After amplification, the PCR products were separated and visualized on a 3% agarose gel.

### Immunohistochemistry

Sections of 5 μm were prepared from formalin-fixed, paraffin-embedded tumors. Immunohistochemical staining was performed using a rabbit anti-human ATM monoclonal antibody (Abcam, Cambridge, UK). For the staining procedure, the DAKO Envision HRP rabbit kit (DAKO, Glastrup, Denmark) with DAB as detection method was used. The scoring of stained section was performed using four grades related to proportion of positive tumor cells: 0: 0%; 1: 1 to 10%; 2: 11 to 50%; 3: > 50%.

### Statistical analyses

Comparisons of the *ATM *mRNA expression levels were performed using the Mann-Whitney rank test (for independent samples). Comparisons of observed mutations between different groups of patients were performed using Fischer exact test. All *P*-values given are two-sided, and for Fischer exact test, cumulative. Multivariate analyses were performed by binary logistic regression, defining *TP53*- and *CHEK2 *mutations as categorical variables and ATM mRNA levels as a continuous variable. Survival analyses were performed by Kaplan-Meier, and subsets of patients were compared using the log-rank test. Deaths for reasons other than breast cancer were treated as censored observations. All statistical analyses were performed using the SPSS 15.0/PASW 17.0 software package (SPSS Inc. Chicago, IL, USA) and/or Simple Interactive Statistical Analysis (SISA).

## Results

### ATM mutations in locally advanced breast cancer

ATM gene alterations recorded through sequencing (*n *= 149 tumors) and MLPA analysis (*n *= 69 tumors) in different breast cancer cohorts are summarized in Table 2 (see Materials and methods for cohort details). Most alterations found were of germline origin and previously observed by others [26-29] in multiple patients, indicating these changes to be polymorphic variants rather than mutations contributing to a malignant phenotype.

**Table 2:** ATM mutations

Mutation	Exon	A. A change	Cohort 1 Dox Fumi *n *(%)	Cohort 2 Epi *n *(%)	Cohort 3 Pac *n *(%)	Germ line	Previous **obs**.^1^
C146G	4	Ser 49 Cys	3 (4.3)	0 (0.0)	1 (2.6)	ND	Yes^2,3,4^
C735T	8	-	2 (2.9)	4 (9.8)	1 (2.6)	Yes	Yes^2,3,4^
A737C	8	Asn 246 Thr	0 (0.0)	1 (2.4)	0 (0.0)	ND	No^5^
C1009T	9	Arg 337 Cys	1 (1.4)	0 (0.0)	0 (0.0)	No	No^5^
A1792G	12	Ile 598 Val	1 (1.4)	0 (0.0)	0 (0.0)	Yes	No^5^
T2572C	18	Phe 858 Leu	2 (2.9)	4 (9.8)	0 (0.0)	Yes	Yes^2,3,4^
C3161G	23	Pro 1054 Arg	5 (7.1)	5 (12.2)	1 (2.6)	Yes	Yes^2,3,4^
A3341G	24	Lys 1114 Arg	0 (0.0)	0 (0.0)	1 (2.6)	Yes	No^5^
C4258T	30	Leu 1420 Phe	3 (4.3)	2 (4.9)	0 (0.0)	Yes	Yes^2,3,4^
T4324C	30	Tyr 1442 His	1 (1.4)	0 (0.0)	0 (0.0)	Yes	No^5^
C4578T	31	-	8 (11.4)	5 (12.2)	9 (23.7)	Yes	Yes^2,3,4^
A5071C	35	Ser 1691 Arg	1 (1.4)	0 (0.0)	0 (0.0)	ND	Yes^2,3,4^
G5557A	38	Asp 1853 Asn	13 (18.6)	9 (22.0)	11 (28.9)	Yes	Yes^2,3,4^
T5793C	40	-	1 (1.4)	1 (2.4)	0 (0.0)	ND	Yes^2,3,4^
C6217G	44	Leu 2073 Val	0 (0.0)	1 (2.4)	0 (0.0)	ND	No^5^
T7390C	51	Cys 2464 Arg	0 (0.0)	0 (0.0)	1 (2.6)	Yes	Yes^2,3,4^
del A 8432	59	Frameshift	0 (0.0)	0 (0.0)	1 (2.6)	ND	No^5^

Overview of the ATM mutations observed in the cohorts of Doxorubicin/Fumi (*n *= 70), Epirubicin (*n *= 41) or Paclitaxel (*n *= 38) treated breast cancer patients.

^1 ^Data from Dörk *et al.*, Thorstenson *et al.*, Bretsky *et al. *and the Leiden Open Variation Database [29]

^1 ^Previously observed in Breast cancer

^3 ^Common variant (considered non-pathogenic)

^4 ^Pathogenic role unclear

Seven of the mutations observed have, to our knowledge, not previously been reported (Table 2). Each of these variants was observed in a single patient only. Peripheral blood lymphocytes were available for four of these patients. Out of these four, one mutation proved to be somatic, whereas three were also found in lymphocyte DNA, indicating the mutations to be of germline origin.

### *ATM *mutations are not associated with chemo-resistance

We compared gene alterations in tumors progressing on therapy (PD) versus tumors not progressing (stable disease or an objective response) classified according to the UICC criteria [30] as previously described [10,31]. The frequency of *ATM *mutations was similar among patients with progressive disease (PD) upon treatment and those responding to therapy in any of the cohorts analyzed (data not shown). Stratifying tumors into wild-type versus *TP53*/*CHK2 *mutated ones did not reveal any imbalance regarding ATM mutation incidence between these subgroups.

Assessing the possibility that some ATM variants in particular could be associated with therapy resistance, we compared individual mutations observed among PD-patients with those observed in responders. However, none of the individual mutations (Ser49Cys, Asp1853Asn, Phe858Leu or Pro1054Arg) was found at higher incidence among patients progressing on therapy as compared to responders.

We further assessed the potential impact of ATM mutations on response to paclitaxel monotherapy. Contrasting what has been recorded for the anthracyclines, *TP53*/*CHEK2 *mutations do not predict therapy resistance towards the taxanes [23]. Five out of the 11 analyzed patients displaying progressive disease upon paclitaxel monotherapy treatment harbored *ATM *mutations (compared to 10 out of 27 responders). Thus, no association between *ATM *mutations and resistance to paclitaxel therapy was recorded.

### Low ATM expression levels predict chemo-resistance to doxorubicin and mitomycin but not to paclitaxel in tumors wild-type for *TP53 *and *CHEK2 *

**Figure 1:**
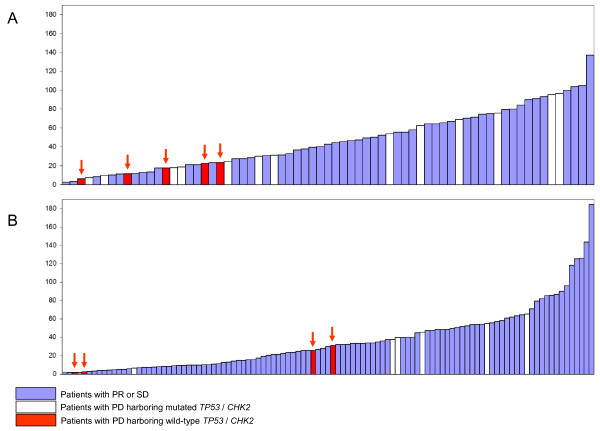
**Relative intratumor ATM mRNA levels among patients with locally advanced breast cancer**. (**A**) ATM mRNA levels among patients receiving neoadjuvant doxorubicin or 5-fluorouracil/mitomycin (*n *= 69; Cohort 1). Blue bars represent patients displaying stable disease or response to treatment. White bars represent patients with progressive disease (PD) and *TP53 *or *CHEK *mutations, while red bars (with arrows) indicate PD-patients with wild-type *TP53 *and *CHEK2*. (**B**) ATM mRNA levels among patients receiving neoadjuvant epirubicin (*n *= 109; Cohort 2). Color key as for A.

ATM mRNA levels were determined by qPCR in 69 out of the 71 doxorubicin or 5-fluorouracil/mitomycin treated patients (Cohort 1) from whom RNA were available (for two patients, one with partial response and one with stable disease upon treatment, sufficient amounts of RNA was lacking; Table 1). Results from these analyses revealed large differences in ATM mRNA levels in the cohort, with a 56.9-fold ratio between the highest and the lowest value recorded (Figure 1A).

No association between ATM mRNA levels and ATM mutation status was observed (*P *> 0.5; Mann-Whitney rank test). Further, since previous studies have indicated that concomitant ATM and p53 inactivation is underrepresented in breast tumors [22], we assessed the ATM levels among *TP53 *mutated versus wild-type tumors; no difference between the two groups was observed (*P *> 0.4).

Considering the 18 patients with progressive disease, these patients displayed a non-significant trend towards lower ATM mRNA levels as compared to the responders (*P *= 0.104; Mann-Whitney rank test), with 12 out of 18 expressing ATM levels below the median value of the cohort (*P *= 0.168; Fischer exact test).

To test the hypothesis that low ATM expression may be an alternative mechanism inactivating the p53 pathway, we compared ATM mRNA expression levels in tumors resistant to chemotherapy despite harboring wild-type *TP53*/*CHEK2 *(Group A Table 3; *n *= 5) to the other tumors (Group B + C + D; *n *= 64) in the same cohort (Cohort 1). ATM-levels were lower among tumors in Group A as compared to the tumors in the other three groups (*P *= 0.012). Stratifying the latter tumors (*n *= 64) into *TP53*/*CHEK2 *mutated (Group B + D; *n *= 40) and *TP53*/*CHEK2 *wild-type (Group C; *n *= 24) tumors, the tumors in the A group expressed lower ATM levels when compared to each of these two subgroups (*P *= 0.010 and 0.028, respectively). Notably, each of the tumors in Group A revealed an ATM expression level in the lower tertile of the total cohort.

**Table 3:** Grouping of tumors used for evaluation of ATM's impact on resistance to chemotherapy

	***TP53/CHEK2 *status**	
	
	**Wild-type**	**Mutated**
	
Progressive disease	A	B
	
Responders	C	D

In contrast, no difference in ATM-levels between PD-tumors harboring *TP53 *or *CHEK2 *mutations (Group B) and the other tumors in this cohort was found (*P *> 0.5); neither did we record any difference in ATM levels between mutated tumors progressing on chemotherapy and mutated tumors responding to treatment (Group B vs. Group D; *P *> 0.5)

In the epirubicin treated validation cohort (*n *= 107, Cohort 2, Table 1), 6 out of 10 patients with progressive disease on therapy previously were found mutated in the *TP53 *or *CHEK2 *genes (Group B) [8], limiting the number of patients with a PD despite harboring wild-type *TP53 *and *CHEK2 *(Group A) to 4. Still, these four patients each revealed low ATM expression levels as compared to the rest of the tumors (*n *= 103; *P *= 0.092; Figure 1B), as well as when compared to the subgroups of other wild-type *TP53*/*CHEK2 *tumors (*n *= 79; *P *= 0.097) or tumors harboring *TP53*/*CHEK2 *mutations (*n *= 25; *P *= 0.094).

To evaluate whether the effect of low ATM status was specific to DNA-damaging chemotherapy, we analyzed the predictive impact of ATM expression levels on response to paclitaxel monotherapy (Cohort 3, Table 1). Here, we observed no difference in ATM expression levels between patients revealing primary resistance to paclitaxel with (*n *= 5) or without (*n *= 7) concomitant *TP53*/*CHEK2 *mutations and patients obtaining an objective response/stable disease (*n *= 102; *P *> 0.2 for both comparisons).

### Low ATM expression levels may substitute for *TP53*/*CHEK2 *mutations as a cause of chemo-resistance

**Figure 2:**
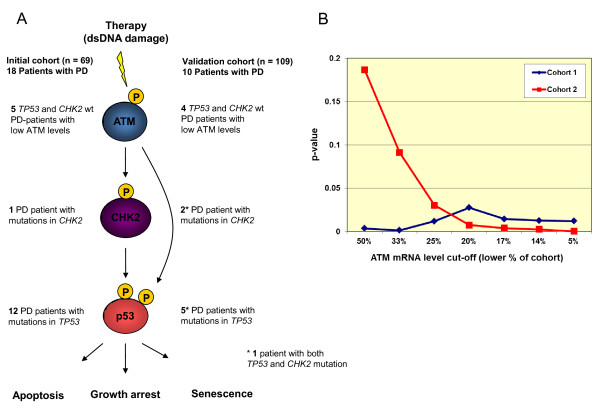
**Alterations in the p53 functional pathway predicts chemoresistance**. (**A**) Schematic illustration of the three central players (ATM, Chk2 and p53), activated in response to chemotherapy induced double stranded DNA breaks. All patients in this study with tumors displaying lack of response to neoadjuvant treatment with doxorubicin or 5-fluorouracil/mitomycin (left) or epirubicin (right) harbor alterations affecting at least one of these factors, leaving the functional pathway disrupted and thus, cells resistant to therapy. (**B**) Graphs displaying *P*-values for the correlations between defects in the p53 functional pathway, defined as low expression of ATM or mutations affecting either *TP53*-L2/L3 domains or *CHEK2*, and lack of response to neoadjuvant treatment with doxorubicin or 5-fluorouracil/mitomycin (blue line) or epirubicin (red line). Percentage on the X-axis indicates the portion of the patients cohorts defined as "low ATM expressors".

Postulating low ATM expression and mutations affecting *TP53 *and *CHEK2 *to be alternative mechanisms inactivating the p53 pathway (Figure 2A), we compared the frequency of tumors having a "hit" in this pathway (either a *TP53 *(L2/L3) or *CHEK2 *mutation or low ATM expression) among chemo-resistant versus tumors responding to anthracycline/mitomycin chemotherapy. Defining low ATM as the levels expressed by the lower 20% percentile of the patients in the cohort, a "hit" in this pathway (either low ATM expression level or a *CHEK2/TP53 *mutation) correlated to therapy resistance (*P *= 0.0267; Table 4). Further, we evaluated the robustness of the model by performing repeated analysis using ATM cut-off values ranging between the 5% and 50% percentile of the cohort defining tumors with "low expression" for ATM (Figure 2B). Notably, the different models all revealed a statistically significant correlation between defects in the p53 pathway (defined as L2/L3-*TP53*/*CHEK2 *mutations or low level ATM expression) and therapy resistance defined as PD on treatment (*P*-values varying from 0.001 to 0.027; Figure 2B). In a multivariate analysis (logistic regression), L2/L3-*TP53*/*CHEK2 *mutations or low level ATM expression was also confirmed to be significantly associated with resistance to therapy (overall test of the model, *P *= 0.010).

**Table 4:** Correlation between alterations in *TP53/CHEK2/ATM *("hit") and *in vivo *resistance to doxorubicin/FUMI

	**Hit**	**Non-Hit**
	
Progressive disease	14^1^	4^2^
	
Responders	23^3^	28^4^
	
		
		*P *= 0.0267

Cohort 1 (Dox/Fumi)

^1 ^Patients lacking response to therapy/*with *mutations in *TP53 *(L2/L3) or *CHEK2 *or low expression of ATM (lower 20% percentile of cohort)

^2 ^Patients lacking response to therapy/*without *mutations in *TP53 *(L2/L3) or *CHEK2 *low expression of ATM (lower 20% percentile of cohort)

^3 ^Patients with response to therapy (SD, PR or CR)/*with *mutations in *TP53 *(L2/L3) or *CHEK2 *or low expression of ATM (lower 20% percentile of cohort)

^4 ^Patients with response to therapy (SD, PR or CR)/*without *mutations in *TP53 *(L2/L3) or *CHEK2 *or low expression of ATM (lower 20% percentile of cohort)

To confirm this observation, similar analyses were performed on a validation cohort of patients having epirubicin monotherapy (Cohort 2, Table 1). We confirmed the observation that low ATM expression levels or mutations affecting either *TP53 *(L2/L3) or *CHEK2 *to be associated with anthracycline resistance (*P *= 0.0074; Table 5). Using different cut-off values classifying between 5% and 50% of the tumors as "low ATM expressors", we confirmed the model to be robust in this cohort as well (*P*-values varying from 0.20 to < 0.01; notably, setting a cut-off between 5% and 25%, all *P*-values were < 0.05; Figure 2B; multivariate analysis: overall test of the model; *P *= 0.007).

**Table 5:** Correlation between alterations in *TP53/CHEK2/ATM *("hit") and *in vivo *resistance to epirubicin

	**Hit**	**Non-Hit**
	
Progressive disease	7^1^	3^2^
	
Responders	25^3^	72^4^
		
		*P *= 0.0074

Cohort 2 (Epirubicin)

^1 ^Patients lacking response to therapy/*with *mutations in *TP53 *(L2/L3) or *CHEK2 *or low expression of ATM (lower 20% percentile of cohort)

^2 ^Patients lacking response to therapy/*without *mutations in *TP53 *(L2/L3) or *CHEK2 *low expression of ATM (lower 20% percentile of cohort)

^3 ^Patients with response to therapy (SD, PR or CR)/*with *mutations in *TP53 *(L2/L3) or *CHEK2 *or low expression of ATM (lower 20% percentile of cohort)

^4 ^Patients with response to therapy (SD, PR or CR)/*without *mutations in *TP53 *(L2/L3) or *CHEK2 *or low expression of ATM (lower 20% percentile of cohort)

### ATM immunostaining

To evaluate whether the differences in ATM mRNA levels were reflected at the protein level, we performed immunohistochemical staining on samples from the initial cohort (Cohort 1, Table 1). While most samples revealing high mRNA levels displayed strong ATM protein staining, interestingly, some samples stained strongly despite expressing low mRNA levels; thus, there was a lack of statistical correlation between ATM mRNA and IHC-staining levels (*P *> 0.5). However, in the group of tumors with progressive disease and wild-type *TP53 *and *CHEK2*, one tumor only was found to stain strongly for ATM.

### ATM gene copy number

Out of the 70 patients from Cohort 1 sequenced for point mutations, material for MLPA-analyses was available from 66 (all included among the 69 analyzed for ATM expression levels). While no larger intragenetic deletions or duplications were observed, 9 out of the 66 tumors harbored a reduced copy number for the entire ATM locus. No association between ATM reduced copy number and either ATM mRNA levels or response to therapy was observed (*P *> 0.2 and *P *> 0.4, respectively).

### ATM promoter mutations and hypermethylations

The observed difference in ATM mRNA levels could be due to different mechanisms of promoter inactivation, including mutations or hypermethylations.

Screening for potential *ATM *promoter hypermethylation in the patients treated with epirubicin (Cohort 2; *n *= 109), none displayed methylation of the *ATM *promoter.

Next, we sequenced the promoter region from position -661 to +105 relative to the transcriptional start site (sequence NT_033899; [32]) in 70 tumors from Cohort 1, no mutations were recorded. The promoter area was found to exist as two distinct haplotypes differing in positions -635 (rs228589) and -10 (rs189037) relative to the transcriptional start site. Homozygosity for the NT_033899 haplotype -635A/-10G was observed in 15 patients (21.4%), 22 patients (31.4%) were homozygous for the -635T/-10A haplotype while 33 patients (47.1%) were heterozygotes. No difference in ATM expression levels between patients harboring the different genotypes were observed (*P *> 0.2; Kruskal-Wallis rank test). This finding was confirmed in Cohort 2 (data not shown).

### Impact of c-myc amplifications

Our data indicate that events other than promoter alterations, like deregulation of trans-acting factors, may be responsible for the alterations in ATM expression levels. N-myc induces expression of miR-421, which in turn suppresses ATM levels [33]. However, while we found the C-myc gene to be amplified in 12 out 69 doxorubicin/FUMI-treated tumors, no correlation between C-myc amplification status and ATM expression levels were recorded (*P *> 0.4; Mann-Whitney test).

### ATM mRNA levels are not associated with breast cancer subclasses

An interesting question is whether low ATM expression may correlate to other tumor characteristics. Among doxorubicin/mitomycin treated tumors analyzed here, 64 have previously been classified according to gene expression profiling [34,35], defining 25 and 11 tumors belonging to the Luminal A and B class, respectively, 12 belonging to the ERBB2+ class, 11 basal-like, while 5 were found belonging to the normal cell-like class. No difference in ATM expression levels between tumors belonging to the different subclasses were recorded (data not shown).

### ATM mRNA levels predict overall survival in breast cancer patients

**Figure 3:**
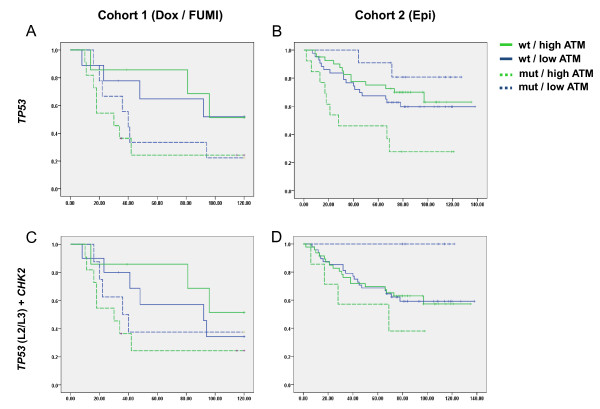
**Impact of ATM expression levels on long-term survival**. (**A**) Kaplan-Meier curves showing long-term survival among breast cancer patients treated with doxorubicin or a combination of 5-fluorouracil and mitomycin in the neoadjuvant setting (Cohort 1), stratified by ATM expression levels (above/below median) and *TP53 *mutation status. (**B**) Data corresponding to (A), but in a cohort of epirubicin treated patients (Cohort 2). (**C, D**) Data corresponding to (A) and (B), but where mutated status is defined as *TP53 *mutation affecting the L2/L3 domains or *CHEK2 *mutation.

Recently, Jiang and colleges suggested low ATM levels on a *TP53 *wild-type background to be associated with poor survival while low ATM predicted improved survival in patients harboring *TP53 *mutations [22]. While we detected a non-significant trend in Cohort 1 (including 69 patients only), we confirmed low ATM levels to predict a poor outcome in patients with tumors wild-type for *TP53 *and *CHEK2 *but to improve long-term outcome among patients with tumors harboring *TP53 *or *CHEK2 *mutations (Figure 3A, C). Further, we observed a significant differential effect of ATM levels on survival pending on *TP53/CHEK2 *mutation status in our larger confirmatory set (Cohort 2; Figure 3B, D; *P *= 0.007; interaction between *TP53 *status and ATM levels on survival *P *= 0.011).

In order to investigate whether these effects on long-term overall survival were of general prognostic nature or related to DNA damaging chemotherapy, we performed a similar analysis on patients enrolled in the paclitaxel treatment arm (Cohort 3). Here, we observed no effect of low ATM levels on prognoses either among patients with tumors wild-type or mutated for *TP53/CHEK2 *(data not shown). This suggests the effect of ATM level on long-term outcome may be related to treatment with DNA-damaging agents.

## Discussion

The mechanisms of resistance to chemotherapy in breast cancer remains poorly understood. While large studies have revealed a somewhat better response related to high Ki67 expression [36] and gene expression profiles, such as a high Oncotype-DX score [37,38], these represent non-specific correlations related to different treatment regimens. While topoisomerase-II amplifications have been associated with improved sensitivity to DNA damaging agents [39], lack of topoisomerase-II expression may not explain anthracycline resistance in general.

We have previously shown mutations in *TP53*, in particular those affecting the DNA binding L2- or L3 domains, to predict resistance to chemotherapy in locally advanced breast cancer [9,10]. Further, exploring the p53 functional pathway, we found mutations in *CHEK2*, the gene coding for the p53 upstream activator Chk2, may substitute for *TP53 *mutations, causing drug resistance in tumors harboring wild-type *TP53 *[8]. Thus, the fact that some tumors harboring wild-type *TP53 *and *CHEK2 *revealed anthracycline resistance made us postulate that these tumors may have defects in other genes involved in the same pathway. While p53 activation in response to DNA damage involves ATM and Chk2 [19,21,26,27], downstream activation of genes leading toward apoptosis or senescence involves multiple, possible redundant, pathways, including transcriptional dependent- as well as independent pathways [40-44]. Thus, we hypothesized inactivation of ATM, but not genes acting downstream of p53, to be a mechanism hampering p53-induced cell death in some tumors.

Contrasting our expectations, mutations in the ATM gene did not correlate to chemo-resistance. Notably, most of the mutations detected are common variants [26-28]. Among novel mutations observed in this study, we confirmed the mutation to be of germline origin in three out of four patients from whom WBC DNA was available. Thus, the mutations observed most likely have little impact on the malignant phenotype of the tumors in which they reside.

In contrast, we observed reduced ATM expression levels in tumors revealing chemo-resistance despite harboring wild-type *TP53*/*CHEK2*, indicating ATM low expression levels may substitute for *TP53*/*CHEK2 *mutations in this respect. Importantly, this result was corroborated by the finding that low ATM expression was associated with a poor long-term outcome for patients with tumors harboring wild-type *TP53*/*CHEK2 *but not among patients harboring *TP53*/*CHEK2 *mutations across the different cohorts. The hypothesis that this finding was due to the effect of DNA-damaging chemotherapy was substantiated by the finding of no effect of ATM expression levels on outcome among patients treated with paclitaxel monotherapy. In contrast, low ATM levels were associated with improved long-term survival in patients treated with anthracyclines/mitomycin harboring *TP53*/*CHEK2 *mutations. This observation is in line with the findings of Jiang *et al. *[22]. These authors found reduced ATM expression to improve outcome in patients with tumors revealing strong staining for p53 (a surrogate for *TP53 *mutations) but a poor prognosis for patients with weak p53 staining (a surrogate marker for wild-type gene status). Because their patients received adjuvant and not primary chemotherapy, direct assessment of chemo-sensitivity could not be applied in their model.

If confirmed in subsequent studies, the findings in the present work have important clinical implications. Given the strong correlation between defects in the ATM-Chk2-p53 pathway and resistance to DNA-damaging therapy, a screen for functional status in this pathway may be a valuable pre-treatment test, indicating whether or not a given patient is likely to respond to such therapy. Patients with an inactive ATM-Chk2-p53 pathway may be spared an unnecessary DNA-damaging treatment and instead given alternative therapy, for example, microtubule poisons.

## Conclusions

Taken together, our data point to p53 and its upstream activators, Chk2 and ATM, as a functional pathway executing response to DNA-damaging chemotherapy in breast cancer patients. If this cascade is disturbed, by *TP53 *or *CHEK2 *mutations or low ATM expression levels, response to therapy may be blunted. Thus, patients harboring tumors with these defects should receive alternative treatment.

## Abbreviations

5FU: 5-fluoro-uracil; ATM: ataxia telangiectasia mutated; CHEK2: checkpoint kinase 2; FUMI: 5-fluorouracil and mitomycin; MSP: methylation-specific PCRs; PD: progressive disease; Ser: serine; SISA: Simple Interactive Statistical Analysis; Thr: threonine; USP: non-methylation-specific PCRs; WBC: white blood cells.

## Competing interests

The authors declare that they have no competing interests.

## Authors' contributions

PEL designed the study and was the PI of all clinical studies. SK and PEL wrote the manuscript. SK, RC and BL performed and supervised laboratory analyses. SK and PEL performed statistical calculations. EL, GA, BØ, SL, TR and IM participated in designing and conducting the epirubicin - paclitaxel study, performed response evaluations, tumor sample collection and management of follow-up data together with PEL. HM performed the immunohistochemistry. All authors approved the final version of the manuscript.

## Supplementary Material

Additional file 1 **Cohorts and primers**. Additional file 1 contains Table S1 with an overview of the patients' cohorts and Table S2 with a list of all primers used. Click here for file
