# Supplementary material for: Low expression levels of ATM may substitute for CHEK2 /TP53 mutations predicting resistance towards anthracycline and mitomycin chemotherapy in breast cancer
Source: Breast Cancer Res. 2012 Mar 15;14(2):R47. doi: 10.1186/bcr3147 (PMC3446381; doi:10.1186/bcr3147)
Supplement: Additional file 1 — Cohorts and primers. Additional file 1 contains Table S1 with an overview of the patients' cohorts and Table S2 with a list of all primers used. [file bcr3147-S1.DOC]

**Table S1.** Patients included in ATM analyses.

| **Cohort** | | | **ATM coding region** | | | **ATM promoter** | |  |
| --- | --- | --- | --- | --- | --- | --- | --- | --- |
| No. | Therapy | Response^1^ | Mutations | mRNA levels | Copy no. | Mutations | Methylation | Survival |
|  |  |  | *n* | *n* | *n* | *n* | *n* | *n* |
|  |  |  |  |  |  |  |  |  |
|  |  | PD^2^ | 17 | 18 | 18 | 17 | - | 18 |
| 1 | Doxorubicin | SD^3^ | 27 | 26 | 26 | 27 | - | 26 |
|  | FUMI | PR^4^ | 26 | 25 | 25 | 26 | - | 25 |
|  |  | CR^5^ | 0 | 0 | 0 | 0 | - | 0 |
|  |  | Total | 70 | 69 | 69 | 70 | - | 69 |
|  |  |  |  |  |  |  |  |  |
|  |  |  |  |  |  |  |  |  |
|  |  | PD^2^ | 10 | 10 | - | 10 | 10 | 10 |
| 2 | Epirubicin | SD^3^ | 12 | 44 | - | 12 | 44 | 44 |
|  |  | PR^4^ | 16 | 50 | - | 16 | 50 | 50 |
|  |  | CR^5^ | 3 | 3 | - | 3 | 3 | 3 |
|  |  | Total | 41 | 109^6^ | - | 41 | 109^6^ | 109^6^ |
|  |  |  |  |  |  |  |  |  |
|  |  |  |  |  |  |  |  |  |
|  |  | PD^2^ | 11 | 12 | - | 11 | - | 12 |
| 3 | Paclitaxel | SD^3^ | 10 | 44 | - | 10 | - | 44 |
|  |  | PR^4^ | 14 | 45 | - | 14 | - | 45 |
|  |  | CR^5^ | 3 | 5 | - | 3 | - | 5 |
|  |  | Total | 38 | 114^7^ | - | 38 | - | 114^7^ |
|  |  |  |  |  |  |  |  |  |

^1^ Clinical response upon neoadjuvant chemotherapy; ^2^ Progressive Disease, ^3^ Stable Disease, ^4^ Partial Response, ^5^ Complete Response.

^6^ Among the 109 patients, two were omitted from statistical analyses as protocol violators.

^7^ Among the 114 patients, 8 had non-evaluable response to therapy.

**Table S2.** Primers and annealing temperature for the PCR-amplification of *ATM* coding exons

| **ATM exon** | **Forward primer** | **Reverse primer** | **Annealing temp.(ºC)** |
| --- | --- | --- | --- |
|  |  |  |  |
| Promoter | GCCTCAAAGGTCCTTCTGTCC | GCACACGACTGAATTTCCCAG | 67.0 |
| 3 | cagaatgtgcctctaattgtacag | ggttactaatcacacttatttcaagG | 55.6 |
| 4 | gatagagctacagaacgaaaggtag | gcaaagataaatgttaagacttacacaC | 55.6 |
| 5 | caacgagtttctgaaattgc | cgacagtaatctgttaagccatT | 60.0 |
| 6 | gccataatttgccaatttcttc | aaaattcacaaacaacaaccttC | 55.6 |
| 7 | aatactgatggagtacttttactatg | aacaaaagaaaaagagattagattaC | 52.0 |
| 8 | ccctgttatacccagttgagc | gacttctatgtttgaatgaagaagC | 55.6 |
| 9 | ctagcagtgtaaacagagtacatac | ctaaatgtgacatgacctacttactG | 52.0 |
| 10 | ctccaacctgggcaacaac | gacactgaatgagaaaatggtaaC | 55.6 |
| 11 | ccttttagtttgttaatgtgatgg | tgtttatctgtaagtcagacataatgC | 51.8 |
| 12 | tgtcctgatagataaagtctttgc | catcaaataagtggagagagcC | 55.6 |
| 13 | gcttttggtcttctaagtgaagc | attaagatgcagctactacccaG | 55.6 |
| 14 | ggcaaagcattaggtacttgg | ctatttctccttcctaacagtttacC | 55.6 |
| 15 | gtagaatttgttcttacaaaagatagag | attagtataaattctcctaccttggC | 55.6 |
| 16 | agtatgtccaagatcaaagtacactg | gacaatcccactgcactcC | 64.0 |
| 17 | tttatttctttgttgcttggttc | attatgcctattagaatcaaaatatG | 55.6 |
| 18 | tgtgcccagcctgattag | agaagtaaaagaaatcccaagtaG | 55.6 |
| 19 | cttctcttagtgttaatgagtgc | catcagataaaatccaagagC | 55.6 |
| 20 | gtaaatgatttgtggataaacctg | aaagaacacacattgctgtgG | 55.6 |
| 21 | tgaacttctgaaaccactatcg | ataggaccacaaaatagactgtacC | 55.6 |
| 22 | gttttctgagtgcttttatcaga | attttcaatggagatcttactaA | 52.0 |
| 23 | aagcagtctttgtttgttaatgag | tgtaagacattctactgccatctG | 55.6 |
| 24 | gctttggaaagtagggtttga | catagcaagcatatgataacagC | 58.0 |
| 25 | ggattagtgagtaggaggtttctg | tatttcacagtgacctaaggaagC | 64.0 |
| 26 | ggtcctactctaaataatattaacaagc | aacaaatttcacatatgtcatG | 54.0 |
| 27 | aagtatgatactttaatgctgatgg | ggttatatctcatatcattcagggA | 56.8 |
| 28 | gagctgtcttgacgttcacag | aaatagacattgaaggtgtcaacC | 64.0 |
| 29 | ttttggaagttcactggtctatg | catatttcaattaatgctgacaaG | 56.8 |
| 30 | aagtgtatttattgtagccgagtatc | aggaagaacaggatagaaagactG | 55.6 |
| 31 | gagatgctgaacaaaaggacttc | aataccattttgaagatgagtcaG | 55.6 |
| 32 | ctaaaagctgggtatcttagacg | attataggcatgagccaatgtG | 56.8 |
| 33 | CAGGCTTAACCAATACGTG | AAGAAACAGGTAGAAATAGCC | 55.6 |
| 34 | AAAAGTGTTGTCTTCATGCTAG | CTATATGTGATCCGCAGTTG | 55.6 |
| 35 | GTATGATCTCTTACCTATGACTCTAC | CTCCATGAATGTCATATTGAG | 56.8 |
| 36 | TTAATAACTGGTGTACTTGATAGG | ATATGCTGGGTATTCAATAAAC | 55.6 |
| 37 | TACAATGATTTCCACTTCTCTT | GATAAACAGGTCATAAACAAGG | 56.8 |
| 38 | ACTCAAACTATTGGGTGGAT | TCCATCTTTCTCTAGAACTGAG | 56.8 |
| 39 | gaatgcctgggactgag | cgtaagaagcaacactcattaca | 55.6 |
| 40 | ccattgtattctatatcaacatgctt | aaccaaaaattctaaattccattac | 55.6 |
| 41 | AGGAGCTTCCAAATAGTATGT | CCCAAAGTATGAGATAAATACTG | 55.6 |
| 42 | GAGTTGGGAGTTACATATTGG | CACACACATAACTCCTTCATAA | 64.0 |
| 43 | TAAACAACGGTATAGTAATTCTG | GTGTAAATATCCACCAACATACT | 56.8 |
| 44 | TCTCTGGTTTTCTGTTGATATC | AAAAAAAATCAAGTCAAATTTC | 54.0 |
| 45 | ATCTTAGGGTTCTGTTTTTAAG | CCATGACATTAAATGTTAAAATAC | 55.0 |
| 46 | CCTCTTCTTTATTTTCAGAGTG | ACAGAAAAGCTGCACTTTAG | 55.6 |
| 47 | gaactctatgtcgtggcattc | gaggtaagatgacatagttttaaattg | 55.6 |
| 48 | GACAAGTAGTTAAGTCCTCAATG | AATAACAGTAAAACACTAATCCAG | 56.8 |
| 49 | tgggtacagtcatggtaatgc | ttaagggttgctccaaaaatC | 55.6 |
| 50 | ttgagtgattctttagatgtatttag | aaattgtcacttgtagaaacctaG | 50.0 |
| 51 | TGTGTGATTTTGTAGTTCTGTTA | ACATCAAGGGGCTTATGTC | 55.6 |
| 52 | CTTACTTGCTTAGATGTGAGAATA | AGAGAGAGAAAATGAGTAACTTATG | 55.6 |
| 53 | ggcatacacgctctacccac | gactgaatatcacacttctaaaagG | 62.1 |
| 54 | TTGGGTAGTTCCTTATGTAATG | ATCTCTACAGAGAGTAACACAGC | 55.6 |
| 55 | GTGGTATCTGCTGACTATTCC | TGACCAATTTTGACCTACATA | 55.6 |
| 56 | aagtgcaaatagtgtatctgacc | gccagagaagggaaggctC | 55.6 |
| 57 | tggtttgagtgccctttgc | cacttcacccaaccaaatgG | 64.0 |
| 58 | aaaaggtatttaatctgtaactcc | cttagtatctttgacaattacctgatG | 55.6 |
| 59 | AGGTAATGTATCCTGTTCATCT | GTGCTCAATCTACTATATGTACAAG | 56.8 |
| 60 | tttctccagTTGGTTACATACTTG | aacaacattccatgatgacC | 55.6 |
| 61 | GCTGTCAAACCTCCTAACTT | GCCCATGTAATTTTGACAT | 55.6 |
| 62 | TTGACAACATTGGTGTGTAAC | CCTGACGAGATACACAGTCTAC | 56.8 |
| 63 | CTGGTTCTACTGTTTCTAAGTATG | CTGAAAAACTGACAACAGGAC | 55.6 |
| 64 | TTTCTTATTCCCAAGGCC | AAAGGCTGAATGAAAGGG | 55.6 |
|  |  |  |  |
